# Loeffler's Endomyocarditis With Embolic Stroke Secondary to Chronic Eosinophilic Leukemia With Therapy-Related Molecular Features

**DOI:** 10.1016/j.jaccas.2026.109890

**Published:** 2026-08-26

**Authors:** Sushmithaa Ramesh, Nadia Yousif, Ziad Affas, John Bajouka, Jiries Haddad, Harsh Thacker, Marcel Zughaib

**Affiliations:** a Department of Internal Medicine, Henry Ford Health Providence Hospital, Southfield, Michigan, USA; b Division of Cardiology, Henry Ford Health Providence Hospital, Southfield, Michigan, USA

**Keywords:** anticoagulation, cardiomyopathy, echocardiography, left ventricle, reduced ejection fraction, thrombus

## Abstract

**Background:**

Loeffler's endomyocarditis is a severe manifestation of hypereosinophilia characterized by eosinophil-mediated myocardial injury, mural thrombus formation, and endomyocardial fibrosis. While often idiopathic, it may arise from clonal eosinophilic disorders carrying higher risk of end-organ damage.

**Case Summary:**

A 77-year-old man with diffuse large B-cell lymphoma in remission presented 5 years after R-CHOP (rituximab, cyclophosphamide, doxorubicin, vincristine, prednisone) chemotherapy with subacute confusion and dyspnea. Echocardiography demonstrated a large apical left ventricular thrombus with mildly reduced systolic function. Cardiac magnetic resonance revealed diffuse subendocardial late gadolinium enhancement. Progressive eosinophilia (peak: 3.07 × 10^9^/L) with cytopenias prompted a bone marrow biopsy, which revealed chronic eosinophilic leukemia with monosomy 7 and pathogenic mutations, raising concern for a therapy-related clonal process. Despite emergent systemic anticoagulation followed by high-dose corticosteroids, brain magnetic resonance imaging showed multifocal embolic infarcts.

**Discussion:**

This case highlights clonal, potentially therapy-related eosinophilia as an under-recognized cause of Loeffler's endomyocarditis, requiring early diagnosis to prevent irreversible fibrosis and embolic complications.

## Visual Summary

**Figure:**
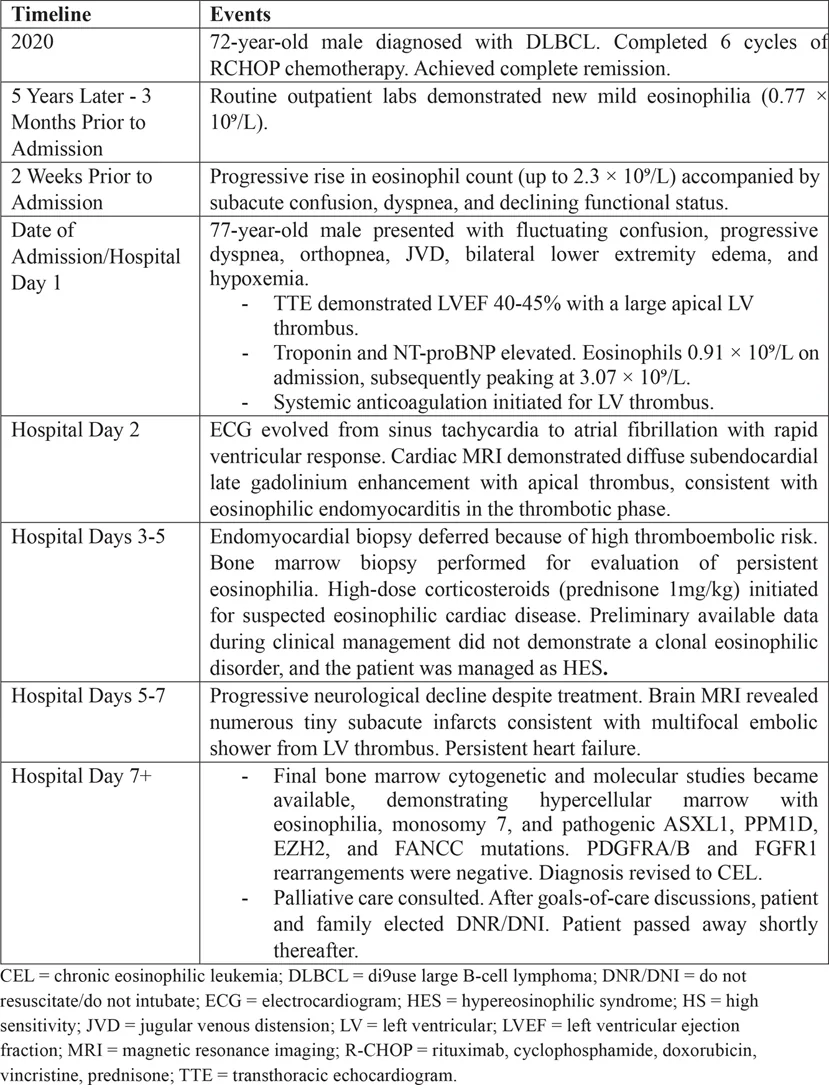

## History of Presentation

A 77-year-old man with diffuse large B-cell lymphoma in remission after 6 cycles of R-CHOP (rituximab, cyclophosphamide, doxorubicin, vincristine, and prednisone) chemotherapy in 2020 presented 5 years after treatment with 2 weeks of fluctuating confusion, progressive dyspnea, and functional decline. His wife reported new dependence in activities of daily living.

In the emergency department, he was tachycardic (heart rate: 100-110 beats/min); hypoxic down to 84%, which improved to 99% on 15 L/min high-flow nasal cannula; and clinically volume overloaded. He had orthopnea, mild jugular venous distension, pulmonary rales/crackles, and bilateral lower extremity pitting edema. Electrocardiogram demonstrated sinus tachycardia with nonspecific ST-segment changes. An acute cardiopulmonary process was initially suspected, and bedside ultrasound revealed a possible left ventricular (LV) mass.

## Past Medical History

The patient had a history of diffuse large B-cell lymphoma treated with 6 cycles of R-CHOP chemotherapy in 2020, currently in remission. Additional history included epilepsy on antiepileptic therapy, asthma treated with Trelegy (fluticasone/umeclidinum/vilanterol), benign prostatic hyperplasia, and osteoporosis.

## Differential Diagnosis

Considerations included the following:

• acute decompensated heart failure with an intracardiac mass (thrombus vs tumor),
• LV thrombus in the setting of cardiomyopathy (ischemic or nonischemic),
• anthracycline-induced cardiomyopathy (late toxicity from prior doxorubicin exposure),
• infiltrative or inflammatory cardiomyopathy (including eosinophilic myocarditis), and
• cardiac malignancy (primary tumor or metastatic disease).

## Investigations

Urgent transthoracic echocardiography with contrast (Figure 1) demonstrated an LV ejection fraction of 40% to 45%, apical hypokinesis, and a large echogenic apical mass. The mass was concerning for intracardiac thrombus formation given its location at the LV apex—the most common site for intracardiac thrombus formation. Its echogenic appearance was consistent with organized fibrin and clot, with its adjacency to a hypokinetic segment, which provides the hemodynamic substrate of blood stasis necessary for thrombus formation.

**Figure 1:**
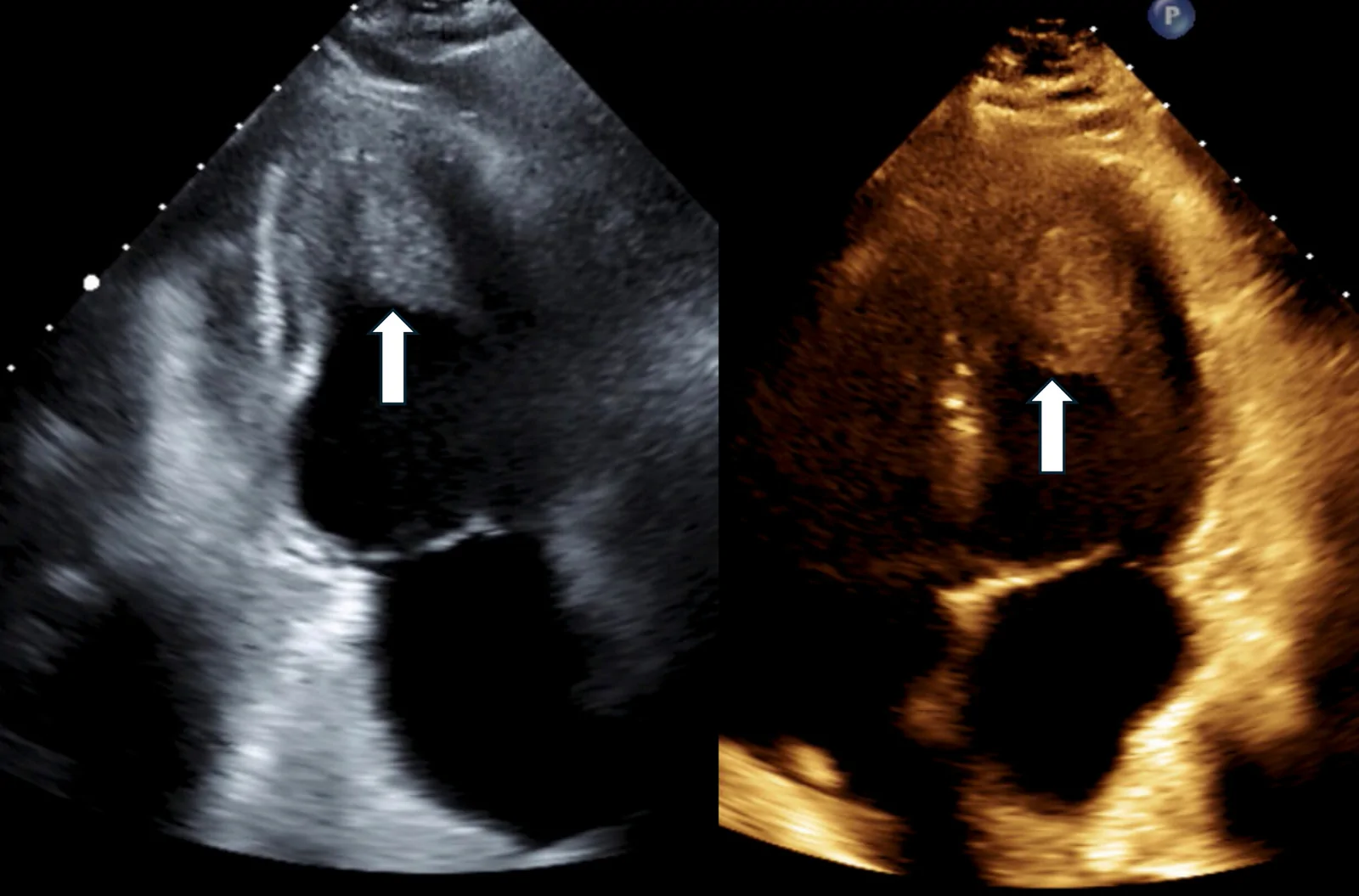
Findings on Contrast-Enhanced Transthoracic Echocardiography Contrast-enhanced transthoracic echocardiography demonstrating mildly reduced left ventricular systolic function (left ventricular ejection fraction: 40%-45%) with apical hypokinesis and a large, well-circumscribed echogenic mass occupying the left ventricular apex (arrows), consistent with mural thrombus.

Laboratory studies were notable for macrocytic anemia (hemoglobin: 9.5 g/dL, mean corpuscular volume: 106 fL), thrombocytopenia (81 K/μL), and eosinophilia that fluctuated initially and then rose rapidly during hospitalization: 0.77 × 10^9^/L (3 months prior), 2.3 × 10^9^/L (2 weeks prior), and 0.91 × 10^9^/L (on admission), with a peak of 3.07 × 10^9^/L.

Prior outpatient evaluation demonstrated persistent cytopenias with eosinophilia, prompting concern for an underlying bone marrow disorder. Peripheral smear revealed mature eosinophilia without blasts.

High-sensitivity troponin increased from 96 to 141 ng/L, and pro-B-type natriuretic peptide was markedly elevated at 19,077 pg/mL, consistent with myocardial injury and heart failure.

Electrocardiogram, which initially demonstrated sinus tachycardia, evolved into atrial fibrillation with rapid ventricular response on hospital day 2.

Cardiac magnetic resonance (CMR) (Figure 2) demonstrated diffuse subendocardial late gadolinium enhancement (LGE) with apical thrombus. Because apical hypokinesis with LV thrombus is classically associated with ischemic cardiomyopathy, ischemic evaluation was considered. However, LGE involved multiple myocardial territories rather than a single coronary distribution, favoring eosinophilic endomyocardial injury over infarction-related scar. With eosinophilia, cytopenias, and high embolic risk from the large thrombus, additional ischemic testing was not pursued, as it was unlikely to change immediate anticoagulation and corticosteroid therapy.

**Figure 2:**
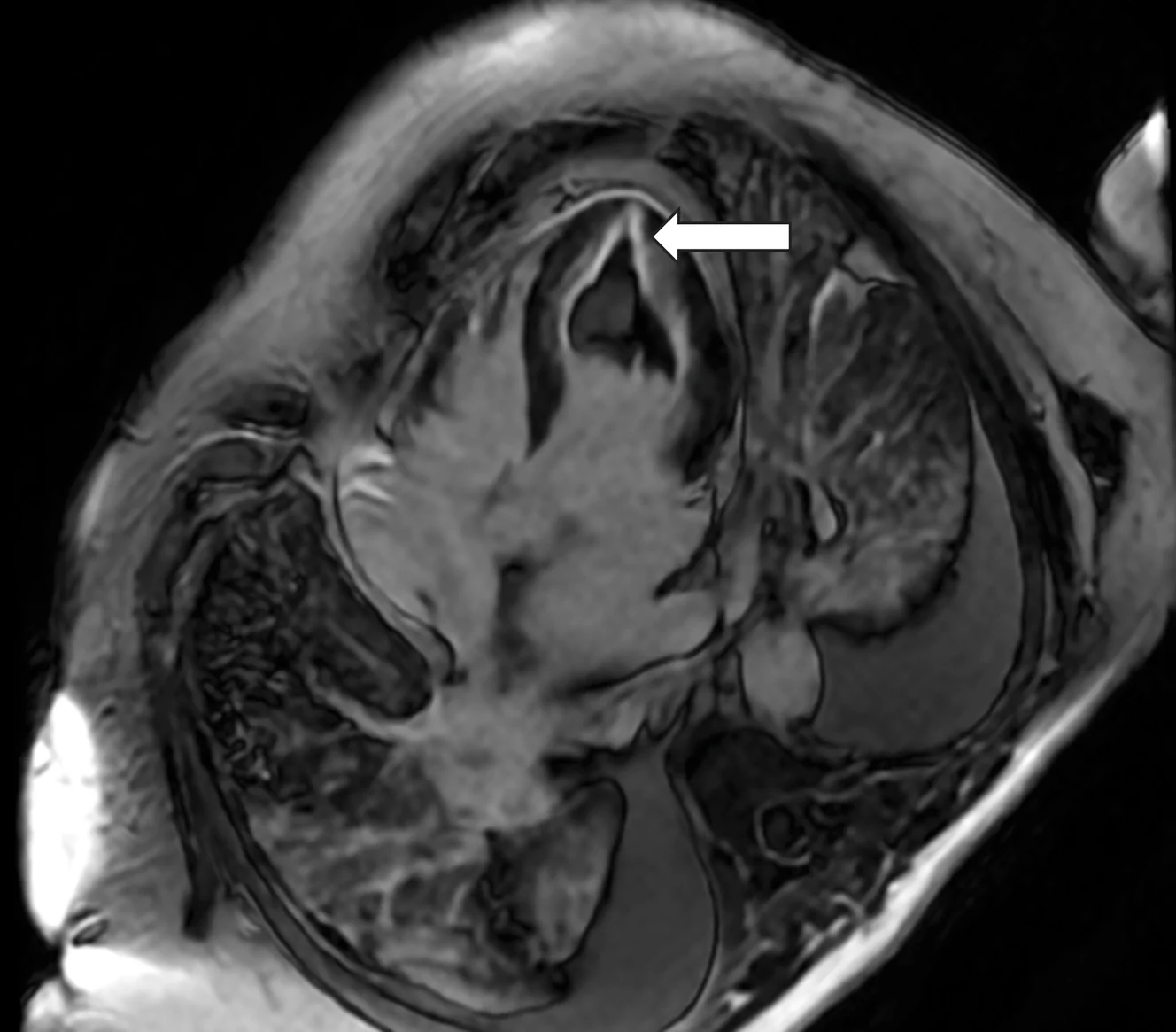
Findings on Cardiac Magnetic Resonance Cardiac magnetic resonance demonstrating diffuse subendocardial late gadolinium enhancement involving the left ventricular apex and midsegments (arrow), consistent with eosinophilic endomyocardial disease.

Serum IgE (856 IU/mL) and interleukin (IL)-5 (23.8 pg/mL) levels were elevated, supporting a cytokine-driven eosinophilic process. Fluctuations in eosinophil count can be attributed to tissue sequestration, diurnal variation, or intermittent eosinophilia patterns in clonal disorders.

Bone marrow biopsy was performed to distinguish a clonal process from idiopathic/reactive eosinophilia; results demonstrated a hypercellular marrow with eosinophilia and cytogenetic abnormalities consistent with a clonal myeloid neoplasm, diagnosed as chronic eosinophilic leukemia (CEL) with monosomy 7. Next-generation sequencing identified pathogenic mutations, including *ASXL1*, *PPM1D*, and *EZH2* alterations, as well as a *FANCC* splice-site mutation. Rearrangements involving *PDGFRA/B* and *FGFR1* were negative. The presence of cytogenetic abnormalities (monosomy 7) and pathogenic somatic mutations established a clonal eosinophilic neoplasm consistent with CEL rather than idiopathic hypereosinophilic syndrome (HES). Although causality cannot be definitively established, the presence of monosomy 7 and *PPM1D* mutation in the setting of prior cytotoxic chemotherapy raised concern for a therapy-related clonal process.

Given the high risk of thromboembolism associated with endomyocardial biopsy in the presence of a large apical thrombus, the procedure was deferred in favor of a noninvasive diagnostic approach.

## Management

Given concern for an intracardiac thrombus, intravenous heparin was initiated on admission. Following bone marrow biopsy, high-dose corticosteroids (prednisone ∼1 mg/kg) were started for suspected eosinophilic cardiac disease, before final cytogenetic and molecular results.

## Outcome and Follow-Up

The patient's eosinophil count decreased from 3.07 × 10^9^/L to 1.45 × 10^9^/L within days of treatment initiation, although prior fluctuations were noted. However, his neurological status progressively worsened, and subsequent brain magnetic resonance imaging revealed numerous tiny subacute infarcts consistent with an embolic shower. Given the extent of neurologic injury and persistent heart failure, palliative care was consulted. After goals-of-care discussions, the patient and family elected do-not-resuscitate/do-not-intubate status, and he passed away shortly thereafter.

## Discussion

Loeffler's endomyocarditis represents a severe cardiac manifestation of hypereosinophilia, defined by persistent eosinophilia ≥1.5 × 10^9^/L with evidence of organ involvement. Cardiac injury progresses through 3 pathophysiologic stages—necrotic, thrombotic, and fibrotic (Figure 3)—ultimately leading to restrictive cardiomyopathy and valvular dysfunction if untreated.^1^^,^^2^ Mortality remains substantial, particularly when diagnosis is delayed to the thrombotic or fibrotic stages.

**Figure 3:**
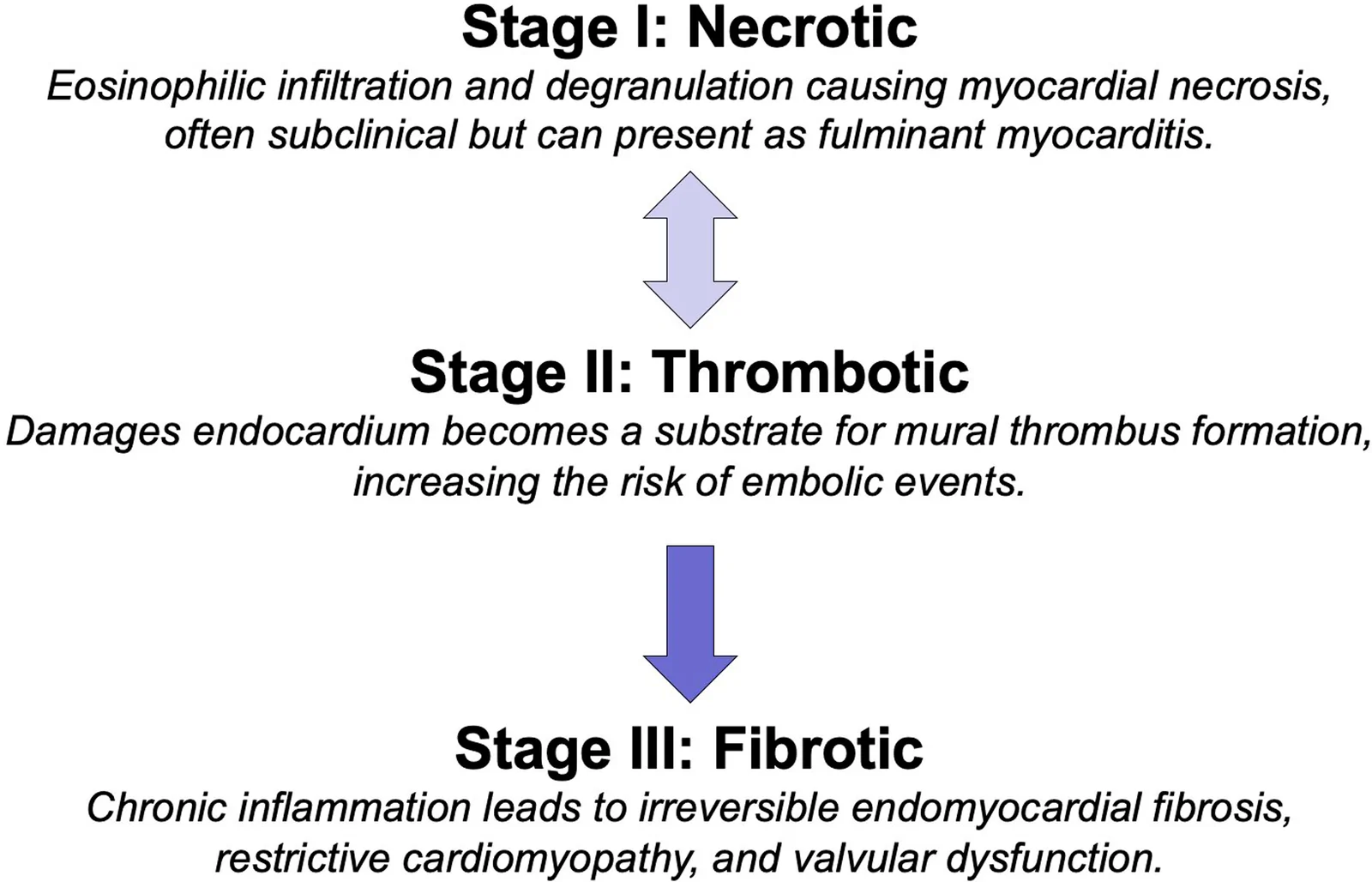
Pathophysiologic Stages of Loeffler's Endomyocarditis Stage I: necrotic eosinophilic injury, stage II: thrombotic mural clot formation, and stage III: fibrotic restrictive cardiomyopathy.

Eosinophilic disorders are classified as reactive, idiopathic, or clonal hematologic processes, the latter including CEL, which is defined by cytogenetic or molecular abnormalities and which carries a higher risk of end-organ damage.^3^^,^^4^ In this case, bone marrow findings demonstrating monosomy 7 and pathogenic mutations (*ASXL1*, *PPM1D*, *EZH2*, *FANCC*) supported a clonal eosinophilic disorder, distinguishing it from idiopathic HES under contemporary classification systems.^4^ These high-risk molecular features, particularly monosomy 7 and *PPM1D* mutation, raised concern for a therapy-related clonal process in the setting of prior R-CHOP chemotherapy exposure. The additional identification of a *FANCC* splice-site mutation suggested possible disruption of the Fanconi anemia DNA repair pathway, which may have contributed to genomic instability and susceptibility to chemotherapy-induced clonal evolution. *PDGFRA*/*B* rearrangements were negative, precluding targeted therapy with imatinib, which achieves a complete hematologic remission in the vast majority of rearrangement-positive cases.^4^ *FGFR1* rearrangement was also absent, excluding pemigatinib as a treatment option.^4^ The absence of all targetable tyrosine kinase gene fusions in this case underscores the limited therapeutic options in rearrangement-negative CEL and the need for novel treatment strategies.

In addition to clonal hematopoiesis, this case highlights the role of cytokine-mediated eosinophilic activation. Markedly elevated IL-5 and IgE levels indicate active Th2-driven signaling, which promotes eosinophil proliferation, activation, and survival. This dual-pathway mechanism—clonal expansion with superimposed cytokine activation—may explain the severity of myocardial involvement, as IL-5–mediated pathways are directly implicated in eosinophil-induced tissue injury.^3^

CMR plays a central role in diagnosis by identifying characteristic diffuse subendocardial LGE and intracavitary thrombus.^5^^,^^6^ Expert consensus recognizes diffuse subendocardial LGE beyond a single coronary territory with LV thrombus as a pattern often seen in eosinophilic myocarditis, supporting distinction from infarction-related LV thrombus.^6^ In this case, CMR favored eosinophilic injury, shifting management away from ischemic revascularization and toward rapid eosinophil suppression with corticosteroids. Embolic complications are well described in the thrombotic stage.^2^^,^^6^

Although R-CHOP contains doxorubicin, a well-established cause of anthracycline cardiomyopathy,^7^ this presentation was not consistent with typical chemotherapy-induced cardiotoxicity. Anthracycline injury usually leads to a progressive dilated cardiomyopathy with diffuse LV dysfunction and no intracavitary thrombus, most often within the first year after treatment, although late-onset cases can occur.^7^ In contrast, this patient demonstrated marked peripheral eosinophilia, a large apical mural thrombus, and diffuse subendocardial enhancement findings characteristic of eosinophilic endomyocardial disease. Distinguishing these entities is critical, as Loeffler's endomyocarditis requires urgent corticosteroid therapy, whereas anthracycline-mediated injury is generally irreversible when diagnosed late and does not respond to immunosuppression.

High-dose corticosteroids are the first line in treatment, typically prednisone 1 mg/kg/d or intravenous methylprednisolone 500 to 1,000 mg daily for 3 days as pulse therapy, with taper guided by eosinophil response and interval imaging. Systemic anticoagulation is essential once thrombus is suspected to reduce embolic complications. Intravenous unfractionated heparin can be used acutely and transitioned to warfarin for long-term therapy. Warfarin has the most established evidence for LV thrombus, though data are largely extrapolated from post–myocardial infarction LV thrombus. Direct oral anticoagulants are increasingly used, with evolving evidence.^8^ Serial imaging is recommended at 2 to 4 weeks and 3 to 6 months to assess thrombus resolution and progression to fibrosis.^2^

Prognosis is highly dependent on the stage at diagnosis. Early recognition during the inflammatory phase may allow reversal of myocardial injury; however, presentation in the thrombotic or fibrotic phase is associated with significantly worse outcomes despite appropriate therapy.^2^ In this case, advanced disease at presentation, compounded by extensive embolic stroke, underscores the narrow therapeutic window and the critical importance of early diagnosis.

## Conclusions

Loeffler's endomyocarditis should be considered in patients presenting with unexplained LV thrombus and peripheral eosinophilia. This case demonstrates that eosinophilic cardiac disease may arise from clonal hematologic disorders such as CEL rather than idiopathic HES, particularly when cytogenetic or molecular abnormalities are present. Prompt recognition and early initiation of corticosteroids and anticoagulation are essential to prevent irreversible endomyocardial fibrosis and embolic complications.

Future studies must clarify optimal steroid duration and the role of targeted biologic therapies such as IL-5 inhibition as well as evaluate long-term outcomes in patients with clonal eosinophilic disorders and cardiac involvement.

## Funding Support and Author Disclosures

The authors have reported that they have no relationships relevant to the contents of this paper to disclose.

**Take-Home Messages**

• A large apical left ventricular thrombus with peripheral eosinophilia should prompt urgent evaluation for eosinophilic endomyocardial disease, including bone marrow biopsy to distinguish clonal disorders such as chronic eosinophilic leukemia from idiopathic hypereosinophilic syndrome.
• Diffuse subendocardial late gadolinium enhancement on cardiac magnetic resonance is highly suggestive of eosinophilic cardiac involvement and should prompt emergent corticosteroids and anticoagulation, even before confirmatory results.

**Visual Summary:** Timeline of the Case

Time	Events
2020	A 72-year-old man was diagnosed with DLBCL. Completed 6 cycles of R-CHOP chemotherapy. Achieved complete remission.
5 y later (3 mo before admission)	Routine outpatient laboratory work-ups demonstrated new mild eosinophilia (0.77 × 10^9^/L).
2 wk before admission	Progressive rise in eosinophil count (up to 2.3 × 10^9^/L) accompanied by subacute confusion, dyspnea, and declining functional status.
Hospital day 1 (admission)	Patient (now age 77) presented with fluctuating confusion, progressive dyspnea, orthopnea, JVD, bilateral lower extremity edema, and hypoxemia. - TTE demonstrated LVEF of 40%-45% with a large apical LV thrombus. - Troponin and NT-proBNP were elevated, eosinophils 0.91 × 10^9^/L on admission, subsequently peaking at 3.07 × 10^9^/L. - Systemic anticoagulation initiated for LV thrombus.
Hospital day 2	ECG evolved from sinus tachycardia to atrial fibrillation with rapid ventricular response. CMR demonstrated diffuse subendocardial LGE with apical thrombus, consistent with eosinophilic endomyocarditis in the thrombotic phase.
Hospital days 3-5	Endomyocardial biopsy deferred because of high thromboembolic risk. Bone marrow biopsy performed for evaluation of persistent eosinophilia. High-dose corticosteroids (prednisone 1 mg/kg) initiated for suspected eosinophilic cardiac disease. Preliminary available data during clinical management did not demonstrate a clonal eosinophilic disorder, and the patient was managed as HES.
Hospital days 5-7	Progressive neurological decline despite treatment. Brain MRI revealed numerous tiny subacute infarcts consistent with multifocal embolic shower from LV thrombus. Persistent heart failure.
Hospital days >7	- Final bone marrow cytogenetic and molecular studies became available, demonstrating hypercellular marrow with eosinophilia, monosomy 7, and pathogenic *ASXL1*, *PPM1D*, *EZH2*, and *FANCC* mutations. *PDGFRA*/*B* and *FGFR1* rearrangements were negative. Diagnosis revised to CEL. - Palliative care consulted. After goals-of-care discussions, patient and family elected DNR/DNI. Patient passed away shortly thereafter.

CEL = chronic eosinophilic leukemia; CMR = cardiac magnetic resonance; DLBCL = diffuse large B-cell lymphoma; DNR/DNI = do not resuscitate/do not intubate; ECG = electrocardiogram; HES = hypereosinophilic syndrome; JVD = jugular venous distension; LGE = late gadolinium enhancement; LV = left ventricular; LVEF = left ventricular ejection fraction; MRI = magnetic resonance imaging; NT-proBNP = N-terminal pro–B-type natriuretic peptide; R-CHOP = rituximab, cyclophosphamide, doxorubicin, vincristine, prednisone; TTE = transthoracic echocardiography.
